# Effectiveness of moving on: an Australian designed generic self-management program for people with a chronic illness

**DOI:** 10.1186/1472-6963-13-90

**Published:** 2013-03-11

**Authors:** Anna M Williams, Leah Bloomfield, Eloise Milthorpe, Diana Aspinall, Karen Filocamo, Therese Wellsmore, Nicholas Manolios, Upali W Jayasinghe, Mark F Harris

**Affiliations:** 1 Centre for Primary Health Care and Equity, University of New South Wales, Sydney, NSW, 2052, Australia; 2 Arthritis NSW, Locked Bag 2216, North Ryde, NSW, 1670, Australia; 3 School of Public Health and Community Medicine, Faculty of Medicine, University of New South Wales, Kensington, NSW, 2052, Australia; 4 Self-Management Support Service, Central Coast Local Health District, Gosford, NSW, 2250, Australia; 5 Rheumatology Department, Westmead Hospital, Sydney West Area Health Service and University of Sydney, Sydney, NSW, Australia

**Keywords:** Self-management, Primary health care, Self-efficacy, Chronic illness

## Abstract

**Background:**

This paper presents the evaluation of “Moving On”, a generic self-management program for people with a chronic illness developed by Arthritis NSW. The program aims to help participants identify their need for behaviour change and acquire the knowledge and skills to implement changes that promote their health and quality of life.

**Method:**

A prospective pragmatic randomised controlled trial involving two group programs in community settings: the intervention program (Moving On) and a control program (light physical activity). Participants were recruited by primary health care providers across the north-west region of metropolitan Sydney, Australia between June 2009 and October 2010. Patient outcomes were self-reported via pre- and post-program surveys completed at the time of enrolment and sixteen weeks after program commencement. Primary outcomes were change in self-efficacy (Self-efficacy for Managing Chronic Disease 6-Item Scale), self-management knowledge and behaviour and perceived health status (Self-Rated Health Scale and the Health Distress Scale).

**Results:**

A total of 388 patient referrals were received, of whom 250 (64.4%) enrolled in the study. Three patients withdrew prior to allocation. 25 block randomisations were performed by a statistician external to the research team: 123 patients were allocated to the intervention program and 124 were allocated to the control program.

97 (78.9%) of the intervention participants commenced their program. The overall attrition rate of 40.5% included withdrawals from the study and both programs. 24.4% of participants withdrew from the intervention program but not the study and 22.6% withdrew from the control program but not the study. A total of 62 patients completed the intervention program and follow-up evaluation survey and 77 patients completed the control program and follow-up evaluation survey.

At 16 weeks follow-up there was no significant difference between intervention and control groups in self-efficacy; however, there was an increase in self-efficacy from baseline to follow-up for the intervention participants (t=−1.948, p=0.028). There were no significant differences in self-rated health or health distress scores between groups at follow-up, with both groups reporting a significant decrease in health distress scores. There was no significant difference between or within groups in self-management knowledge and stage of change of behaviours at follow-up. Intervention group attenders had significantly higher physical activity (t=−4.053, p=0.000) and nutrition scores (t=2.315, p= 0.01) at follow-up; however, these did not remain significant after adjustment for covariates. At follow-up, significantly more participants in the control group (20.8%) indicated that they did not have a self-management plan compared to those in the intervention group (8.8%) (X^2^=4.671, p=0.031). There were no significant changes in other self-management knowledge areas and behaviours after adjusting for covariates at follow-up.

**Conclusions:**

The study produced mixed findings. Differences between groups as allocated were diluted by the high proportion of patients not completing the program. Further monitoring and evaluation are needed of the impact and cost effectiveness of the program.

**Trial registration:**

Australian New Zealand Clinical Trials Registry: ACTRN12609000298213

## Background

In 2005, chronic illnesses were responsible for 35 million deaths worldwide. Chronic conditions such as diabetes, heart disease, chronic respiratory disease, stroke and cancer account for the greatest mortality and morbidity internationally [1]. In Australia, coronary heart disease, anxiety and depression are the major sources of disease burden and cardiovascular, cancer and respiratory diseases cause the greatest mortality [1]. 77% of the population has a chronic illness [1] and approximately 55% of people aged between 65-84years have five or more chronic illnesses [2].

These high rates of mortality and morbidity and associated health care costs have led to an increasing focus on the role of patients in making decisions and managing their chronic illnesses [3,4] and the role of health care providers in providing self-management support.

Since the 1980s a wide range of self-management support initiatives have been developed internationally, including care and action plans, health coaching, self-help groups, disease-specific and generic self-management education programs for both individuals and groups. There are two widely known generic chronic disease self-management group education programs: the Stanford Chronic Disease Self-management Program (CDSMP) and the Expert Patient Program (based on the Stanford CDSMP).

Both programs are based on the theory of self-efficacy and consist of a six-week group education program (2.5 hour sessions each week) delivered by two trained lay leaders who implement the program according to a leader’s manual. The range of topics is broadly similar in the two programs: exercise, symptom management, diet/nutrition, fatigue and pain management, medications, community resources, managing fear, anger and depression, problem solving and communication with health care providers. Participants produce weekly action plans and set self-management goals, with support from the leaders and fellow participants.

The Stanford program has been disseminated widely in the US, UK, Australia and Asia. It has been found effective in improving health outcomes and reducing costs for patients with different chronic illnesses [5,6], although the reported effect sizes are small [7].

Participation in the Expert Patient Program is based on the patient’s understanding of the nature of chronic illness and does not require diagnosis or referral from a health care provider. Implemented across the UK, the program is reported to be effective in improving patient self-efficacy, energy and health-related quality of life. There is a small effect on reducing the cost to patients of health and allied services and medications and there is no reported influence on health service utilisation rates [8].

In Australia, self-management initiatives have been supported by the Department of Health and Ageing since the late 1990s. These have included the $36.2 million Sharing Health Care Initiative (SHCI) in 2002-03 [9] and activities under the Council of Australian Governments’ (COAG) Better Health Initiative, for example, the National Chronic Disease Strategy [10]. Some SHCI Demonstration Projects used the Stanford program; however, ongoing use of the program is limited due to costs associated with licensing and training personnel to deliver the program.

Arthritis NSW, therefore, identified the need for a generic self-management group education program that was relevant to the Australian health care system. This led to the development of “Moving On – a self-management program for people with a chronic illness” [11].

This paper presents the evaluation of the Moving On program. Primary hypotheses were that, compared to control group, participants in the intervention group would show a greater improvement in:

1. self-efficacy;

2. self-management intentions and reported behaviours and lifestyle behaviours;

3. health status.

Secondary hypotheses were that intervention program participants would show greater improvement in scores for work and social adjustment, anxiety and depression, greater knowledge concerning use of medicines and more appropriate use of health services.

## Methods

### Study design

The evaluation was a prospective pragmatic randomised controlled trial (RCT) comparing patients’ self-reports following participation in the Moving On intervention program and in a control program of similar intensity (Table 1).

**Table 1:** Description of the intervention and control study groups

	
Intervention program: Moving On	The aim of Moving On is to build participants’ confidence and skills to self-manage their chronic condition/s, using group education and individual self-management plans. The program is based on the theory of self-efficacy [12] and the Trans-theoretical Behaviour Change Model [13]. It addresses behaviour change across a continuum from a pre-contemplation state to behaviour maintenance and recognises that the behaviour changes are often bi-directional and need to be made incrementally, reviewed regularly and supported.
	Moving On consists of seven modules (one 3-hour session per week for seven consecutive weeks) delivered by two trained facilitators, a health professional and a lay leader. An introductory session is followed by six sessions covering: managing fatigue, physical activity, healthy eating, leisure, coping with a chronic illness, stress management, relaxation, getting a good night’s sleep, getting the most out of your medicines, working with your health care team and putting it all together -developing personal action plans or self-management review and evaluation. Participants receive a workbook and reading material for each module and are encouraged to develop goals relating to the different modules. Weekly reviews are built in to each session. During the final session participants are encouraged to develop a plan to continue self-management after the end of the program. A copy of the final self-management plan is sent to their referring primary health care provider and/or general practitioner, with the participant’s consent. Group discussions and sharing of experiences and management techniques are used rather than more didactic methods.
	Moving On differs from the Stanford Program and the Expert Patient Program in that it is based on the theories of self-efficacy and the Trans-theoretical Behaviour Change Model. In addition, Moving On uses a trained health care professional in addition to a lay leader to run the programs. Through sharing the self-management plan developed in the final session of the program, Moving On also promotes a link between the self-management program and the patients’ ongoing primary health care provider(s), thus supporting continuity of care.
Control program: light physical activity	The control program was a previously evaluated light physical activity program delivered by a trained fitness leader for one 1-hour session per week for 7 weeks [9]. This was designed for people with long-term health conditions and incorporates gentle aerobic activity, stretching and muscle strengthening. Exercises undertaken during the sessions are individualised for participants so as to take into account their health and extent of physical activity that is appropriate for them. The light physical activity program is appropriate for persons who may not have previously engaged in exercise.

Semi-structured interviews were also conducted by telephone with a sample of participants, program leaders and referring health care providers. The interviews were conducted after participants had completed their follow-up surveys to ascertain their views on the study and the usefulness of the intervention program. The findings of the qualitative component will be reported elsewhere.

Prior to conducting the RCT, two feasibility studies were conducted: a pre-test of the intervention program modules in 2007 and a pilot study in 2008 to test the implementation and quality assurance procedures of the intervention program and the research methods to be used in the RCT [12].

### Ethics

Ethics approval to conduct the study was obtained from the Human Research Ethics Committee of the Sydney West Area Health Service acting as a lead Ethics Committee for multi-site research within NSW Health Services; this approval was ratified by the Human Research Ethics Committee at the University of New South Wales.

### Participants

Participants were assessed for eligibility and referred to the study by a health care professional. Allocation to programs occurred after enrolment. Participants were required (1) to be aged 45-75yrs; (2) to have a chronic illness diagnosed more than 6 months previously; (3) to have a good understanding of the medical management of their illness; (4) to speak, understand and write English; (5) to agree to comply with session attendance requirements; and (6) to be able to participate in light physical activity. Patients who had a diagnosis of a mental illness (other than co-morbid anxiety or depression), cognitive impairment including dementia or substance abuse, or were in the late stage of palliative care were excluded.

The upper age limit of 75 years was intended to help exclude people who were less likely to benefit from attending a self-management program due to an undiagnosed cognitive impairment or the diminishing ability to acquire new information with age, particularly for people with co-morbidities [13].

### Recruitment

Patients were recruited by primary health care providers (GPs, private and public allied health care providers, community health nurses) across the north-west of the Sydney metropolitan area between June 2009 and October 2010. In a small number of cases (n= 34), partners of enrolled participants were also referred. The primary health care providers were briefed individually about the aims of the study, the referral and feedback processes. The referral form required patients to provide written consent for their contact details to be forwarded to the University to enrol in the study. The study was also promoted widely within the targeted area by means of brochures in primary health care provider waiting rooms, billboards, press releases to local radio and other news media and presentations to community groups. A 1800 free-call centre was set up to handle responses to the promotion, including distribution of the referral brochure with instructions on how to join the study.

Patients who were referred were sent a study package containing a letter introducing the study, an information sheet, a consent form and a baseline survey. The study package explained that if interested in participating, they should return the completed consent form and baseline survey to the university in the addressed pre-paid envelope. Following the return of their consent form and baseline survey they were to be notified which program they had been allocated to (Moving On or the control program) and the time and location of their program. Patients were invited to contact a member of the research team if they had any questions.

After two weeks, if the consent form and survey had not been returned, patients received a follow- up call. Addresses were checked and an additional package sent where necessary. Referred patients not wishing to participate in the study were asked to provide their age and details of their chronic illness and were thanked for their initial interest in the study.

### Sample size

Initial sample size calculations were based on the primary outcome of change in self-efficacy on data from the 6-item Chronic Disease Self-Efficacy Scale [14] for power of 80% and precision of 95% to detect a 10% difference in the mean self-efficacy scores between the two groups (standard deviation of 2.2, range 1–10 and mean 5.17). These calculations suggested that 225 participants per group would be required. This target was reviewed against the study budget and time frame and a final sample size target of 300 was agreed, with 150 participants in each study arm. This number took into account a predicted 20% loss to follow-up or withdrawal, based on evidence in the literature.

Post hoc sample size calculations were undertaken when the study failed to reach the targeted sample size of 300. The post hoc sample size calculations for power of 80% and precision of 95% to detect a 16% difference in the mean group self-efficacy score of 5.17 (SD = 2.2) indicated a minimum sample of 88 participants in each group. For other variables the sample size was between 63 and 96 participants.

### Randomisation procedure and allocation to programs

Randomisation occurred throughout the enrolment period of the study. When at least six enrolments were received the participants’ ID numbers were sent for block randomisation, in an MS Excel worksheet, to a statistician (UJ) who was not otherwise involved in the study. The study coordinator recorded the allocation in the evaluation database and notified participants by telephone and letter of the details of their program, including dates, time, venue, leader’s name and contact number. Participants could choose different locations or times of their program but not which program they were allocated.

#### Measures

Patient outcomes were self-reported via pre- and post-program surveys using both previously validated tools and questions/measures developed specifically for the study. The surveys were mailed to participants at the time of enrolment and 16 weeks after the commencement of their program. Demographic data obtained included gender, age, main chronic illness, employment status and accommodation type. Primary outcome measures were self-efficacy, self-management knowledge and behaviour and perceived health status. Secondary outcome measures were work and social adjustment, anxiety and depression and appropriateness of health service use.

#### Instruments - primary hypotheses

##### Self-efficacy

Self-efficacy was measured using the Self-Efficacy for Managing Chronic Disease 6-Item Scale [14]. The scale consists of six questions scored on a Likert scale from 1–10 (‘not at all confident’ to ‘totally confident’). The scale score was the mean of the six items. Higher numbers indicate higher self-efficacy.

##### Self-management knowledge and lifestyle behaviours

Questions/measures used to assess changes in participants’ self-management knowledge and lifestyle behaviours were largely developed for the study and were as follows:

a. Existence of a self-management or action plan for chronic illness, measured by a single categorical yes/no question;

b. Change in self-management knowledge. A set of questions devised for the study measured self-reported stage of change in adopting new ways to manage aspects of their chronic illness, including fatigue, physical activity, eating a balanced diet, coping with their chronic condition, managing stress or relaxing, improving sleep routine, communicating with health care professionals and managing medicines. The stage of change was rated using Prochaska and DiClemente’s Stages of Change Model (pre-contemplation, taking action and behaviour maintenance) [15];

c. Sharing a self-management or action plan with a primary care provider. Participants in the Moving On Program completed a set of yes/no questions devised for the study at the end of the final session, indicating:

i. willingness for the program coordinators to forward a copy of the action or self-management plan developed during the study to their general practitioner;

ii. willingness for the program coordinators to forward a copy of the action or self-management plan developed during the study to the health care provider who referred them to the study (in cases where this was not the patients GP); and

iii. intention to take their action or self-management plan on their next visit to their general practitioner.

d. Changes in self-management behaviours were reflected by the following:

i. development or use of a self-management plan: Two questions devised for the purpose of the study measured participants’ self-reported stage of change (Prochaska and DiClemente’s Stages of Change Model) in developing or updating a self-management/action plan and discussing the plan with their GP;

ii. intention to make lifestyle changes: a set of questions, devised for the purpose of the study, measured participants’ self-reported stage of change (Prochaska and DiClemente’s Stages of Change Model) in relation to smoking, nutrition, alcohol and physical activity;

iii. reported lifestyle changes: measured by a validated tool [16] in which participants reported their frequency of light/moderate physical exercise and consumption of fruit, vegetables, dietary fat, alcohol and cigarette smoking. Mean scores were computed for diet, physical activity and alcohol consumption. Higher scores indicated greater levels of physical activity and increased fruit and vegetable intake. For alcohol consumption, lower scores indicated a reduction in consumption;

iv. use of a medicines list: measured by a single categorical Yes/No/NA question devised for the study; and

v. seeking information about medicines: measured by seven questions devised for the purpose of the study, about participants discussing their medicines with a doctor and pharmacist, disclosing non-prescription medicines, seeking a medicine leaflet and other information pertaining to their medicines.

All questions using stage of change measures were subjected to factor analysis in order to reduce complexity (see later section on statistical analysis).

##### Health status

Health status was measured using the Self-Rated Health Scale [17] and the Health Distress Scale [18]. The self-rated health scale consists of one question scored on a Likert scale from 1–5 (‘excellent’ to ‘poor’). The Health Distress Scale consists of four questions concerning health status in the past month, scored on a Likert Scale from 0–5 (‘none of the time’ to ‘all of the time’).

#### Instruments - secondary hypotheses

##### Work and social adjustment

The Work and Social Adjustment Scale was used with permission [19]. The scale consists of six questions scored on a Likert scale from 0–8 (‘not at all’ to ‘very severely’). The final score is the sum of the items, a higher score indicating poorer work and social adjustment.

##### Anxiety and depression

Anxiety and depression were measured using the Hospital Anxiety and Depression Scale (HADS) with permission [20]. The scale consists of four questions for each component. Responses are scored as normal, borderline abnormal and abnormal. Higher scores indicate poorer health.

##### Appropriateness of health service use

Appropriateness of health service use was measured by a validated tool [16] which included the frequency of presentations to emergency department, hospital outpatient department, GP or community health centre in the previous 4 months and type of clinician seen.

#### Intervention program fidelity

The fidelity of the intervention program implementation was monitored using a checklist devised specifically for the purposes of the study. The checklist examined the delivery of the content of the modules including whether module objectives were met, whether key topics were covered and whether module related activities such as goal setting and action or self-management planning were achieved. Fidelity monitoring also reviewed the way in which leaders presented and facilitated the modules, specifically in relation to being well-prepared to run the sessions, creating a supportive environment and facilitating discussion.

Each program was subjected to two integrity checks undertaken by an independent observer using a standard checklist. For each program, the final session (session seven) received an integrity check and the other session was chosen at random. The checklist were analysed descriptively.

##### Statistical analyses

Data were analysed on an intention to treat basis for all participants who completed the follow-up survey. This included those who completed the program and those who withdrew. Descriptive statistics were used for the demographic and baseline characteristics of participants. Univariate analyses (t-tests, Chi-Square, Fisher’s Exact and McNemar) were performed to compare demographic characteristics, baseline and follow-up scores within and between the intervention and control groups. One-tailed tests were carried out in view of the directionality of the research hypotheses, with an alpha of 0.05.

Multivariate analyses using linear and logistic regression were performed for findings that were found to be significant in the univariate analysis, adjusting for age, gender, duration of main illness, program allocation and country of birth. The findings of the multivariate analyses are presented in Table 2.

**Table 2:** Multivariate analyses findings: factors associated with dependent variables assessed by linear and logistic regression

**Dependent variables (4 months follow-up)**									
**Covariates (Reference)**	**Self-efficacy B (SE) p-value**	**Health distress B (SE) p-value**	**Depression score B (SE) p-value**	**Anxiety score B (SE) p-value**	**Work & social adjustment B (SE) p-value**	**Diet scores B (SE) p-value**	**Physical activity B (SE) p-value**	**Medicines list B (SE) p-value**	**Visits to outpatient department B (SE) p-value**
Age 45–54 (>65)	-.048 (.392) .902	.126 (.207) .544	-.441 (.618) .478	-.073 (.620) .907	−2.299 (1.648) .166	-.409 (.428) .342	-.264 (.210) .212	-.437 (.845) .605	-.166 (.731) .874
Age 55–64 (>65)	-.107 (.306) .728	-.008 (.163) .962	-.167 (.482) .729	.450 (.493) .363	.271 (1.352) .842	-.237 (.340) .487	-.310 (.169) .070*	-.227 (.695).744	-.206 (.584) .725
Female (Male)	.361 (.295) .224	.003 (.159) .985	.371 (.467) .428	.486 (.495) .329	1.382 (1.279) .283	.246 (.335) .466	-.179 (.161) .269	-.248 (.679) .714	.341 (.559) .541
Intervention (Control)	.099 (.273) .718	-.202 (.144) .165	.973 (.422) .023	.502 (.439) .255	1.054 (1.178) .373	.260 (.308) .399	.005 (.146) .974	.764 (.609) .210	-.020 (.537) .970
Total no. chronic illnesses	-.006 (.013) .645	.003 (.007) .657	.012 (.021) .577	-.016 (.022) .451	-.014 (.057) .802	.005 (.015) .716	-.007 (.007) .354	.139 (.248) .577	.027 (.175) .879
Australian born (Other)	-.191 (.286) .505	.125 (.153) .414	-.008 (.450) .985	-.502 (.460) .278	−1.227 (1.235) .323	.141 (.316) .656	-.189 (.156) .228	1.161 (.710) .102	-.013 (.553) .981
Baseline outcome score	.164 (.084) .053	.316 (.079) .000	388 (.060) .000	.437 (.058) .000	630 (.067) .000	.610 (.087) .000	.499 (.070) .000		

Note: B = Beta value, SE = Standard error, * = approaching significance.

Principal component factor analysis with varimax rotation was conducted for the 14 items measuring self-management behaviours and lifestyle behaviours associated with the stages of change. The number of factors was determined using the scree test and eigen value > 1. Two component factors emerged: (1) new self-management behaviours and (2) lifestyle behaviours. The factor means, based on scale scores of 0 to 3, were 2.145 (SD= 0.970) and 2.431 (SD= 0.963) respectively (Table 3).

**Table 3:** **Participant readiness to change self-management and lifestyle behaviours and rotated factor loadings for items included in the factor analysis**^**a**^

**Sub-scale items**	**No. responded**	**% (N)**	**Factor load**	
			**RTC SM behaviours**	**RTC Lifestyle Risk Factors**
**RTC SM behaviours**				
Would you take any of the following actions to change your lifestyle?				
Use new ways to manage fatigue	234	93.6 (250)	**.770**	.183
Use new ways to increase my physical activity	246	98.4 (250)	**.515**	.390
Use new ways to cope with my chronic condition	235	94.0 (250)	**.685**	.253
Use new ways to manage stress and/or relax	242	96.8 (250)	**.749**	.202
Use new ways to improve my sleep routine	242	96.8 (250)	**.803**	.185
Use new ways to communicate with my health care professionals	238	95.2 (250)	**.771**	.187
Use new ways to help me manage my medicines better	233	93.2 (250)	**.746**	.135
Get more from my doctors’ appointments by discussing my self-management plan	202	80.8 (250)	**.728**	.217
Make or update my self-management plan or action plan to meet lifestyle needs	195	78.0 (250)	**.774**	.248
**RTC Lifestyle Risk Factors**				
Indicate your plans for the following lifestyle changes:				
Eat more fruits or vegetables	242	96.8 (250)	.174	**.841**
Eat less dietary fat	242	96.8 (250)	.154	**.864**
Do more physical activity	242	96.8 (250)	.302	**.705**

Note: Principal axis factor analysis, rotated using the varimax rotation and the number of factors was determined using the screen test and Eigen value > 1.

Component factor 1, new self-management behaviours, combined the scores for participants’ stage of change associated with using new ways to manage fatigue, cope with a chronic condition, relax and manage stress, improve sleep routines, manage medicines, develop or update a self-management or action plan, and discuss the plan with a GP. Component factor 2, lifestyle behaviours, combined scores for stage of change in eating more fruit and vegetables, eating less dietary fat and increasing physical activity. Univariate analysis was performed, comparing baseline and follow-up scores for the two factors between and within the intervention and control groups.

All analyses were performed using SPSS (version 18.0; SPSS, Chicago, IL, USA).

## Results

### Referral and enrolment of participants

**Figure 1:**
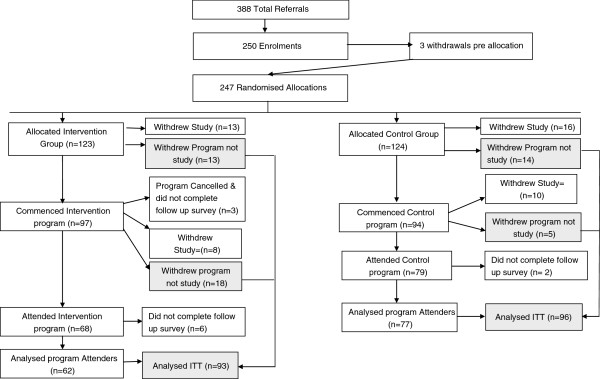
Flow diagram of progress through two arms of the trial showing study and program attrition rates.

Altogether 388 referrals were received of which 250 (64.4%) enrolled in the study (Figure 1). Feedback from referring health care providers and patients suggested that non-enrolment was largely due to competing commitments (including caring for a partner, upcoming travel, employment and pre-booked hospitalisation), poor health status or lack of choice around allocation to the programs. No significant difference was found between enrolling and non-enrolling participants in relation to age, gender, type and duration of the main illness or socio-economic status based on place of residence.

### Allocation to study programs and attrition

A total of 22 programs (12 intervention and 10 control) were conducted between September 2009 and October 2010. In all, 25 block randomisations were performed with 123 participants randomly allocated to the intervention and 124 to the control program. An additional three participants enrolled in the study but withdrew prior to allocation and a total of 100 (40.5%) participants withdrew during the study (Figure 1).

Of the 123 people allocated to the intervention arm, 97 (78.9%) commenced the program of whom 62 (50.4%) attended the intervention sessions and completed the follow-up survey. These are referred to as “intervention program attenders”. A total of 55 people withdrew (44.7%) of whom 24 (19.5%) withdrew from the study and 31 (25.2%) withdrew from the intervention but remained in the study and completed the follow-up survey. They were included in the intention to treat analysis with the program attenders.

Of the 124 people allocated to the control program, 97 (78.9%) commenced the program of whom 77 (62.1%) attended the control program sessions and completed the follow-up survey. These are referred to as “control program attenders”. A total of 47 people withdrew (37.9%) of whom 26 (20.9%) withdrew from the study and 19 (15.3%) withdrew from the control program but remained in the study and completed the follow-up survey. They were included in the intention to treat analysis with the program attenders.

There were no significant differences in gender, age, main health condition or socio-economic status between participants who completed the programs and those who withdrew, whether in the intervention or control program. Withdrawals from 8 of the 12 intervention programs and 7 of the 10 control programs occurred due to ill health, competing life priorities, dislike of the program or, in the case of the intervention, lack of interest in a group education program.

### Intervention program fidelity

A total of 24 integrity checks were performed during implementation of the 12 Moving On Programs by a total of five independent observers. One observer performed 18 (75%) of the checks. The results of the integrity checks suggest that each session of the Program that was audited was delivered largely in line with the Program Guidelines. The audit process also highlighted areas for further development or modification as part of ongoing quality assurance. These included the length of time spent on each activity, the use of the data projector and additional leader training in facilitating group discussions and supporting participants to develop a self-management plan.

### Participant characteristics

Most study participants were female (65.1%) aged 55–74 yrs (78.2%), born in Australia (56.0%), spoke English at home (86.7%), lived in their own home (82.4%), resided in local government areas with the highest socio-economic decile (67.5%) and were either currently employed or retired (76.1%) (Table 4). The most frequent health conditions reported were diabetes (21.1%), osteoarthritis or rheumatoid arthritis (19.0%) and circulatory diseases including heart disease, hypercholesterolemia and hypertension (13.4%). With regard to the number of chronic illnesses, 59 (23.9%) participants reported having one, 62 (25.1%) reported two and 120 (48.5%) reported having 3 or more.

**Table 4:** Participant characteristics at baseline and follow up

**Characteristic**	**Moving on baseline (N=123)**	**Moving on 4 month follow-up (N=93)**	**Control group baseline (N= 124)**	**Control group 4 month follow-up (N=96)**
	**N (%)**	**N (%)**	**N (%)**	**N (%)**
**Gender**				
Female	80 (65)	60 (64.5)	79 (63.7)	63 (65.6)
Male	43 (35)	33 (35.5)	45 (36.3)	33 (34.4)
**Age (Yrs)**				
45-54	28 (23.0)	20 (21.5)	26 (21.5)	15 (15.6)
55-64	52 (42.6)	35 (37.6)	50 (41.3)	37 (38.5)
65-74	38 (31.1)	33 (35.5)	44 (36.4)	42 (43.8)
75+	4 (3.3)	4 (4.3)	1 (0.8)	2 (2.1)
**Speak english at home**				
Yes	109 (89.3)	82 (88.2)	104 (86.0)	81 (84.4)
No	13 (10.7)	11 (11.8)	17 (14.0)	14 (14.6)
**Country of birth**				
Australia	74 (61.2)	61 (65.6)	60 (48.8)	46 (47.9)
Other country	47 (38.8)	30 (32.3)	63 (51.2)	48 (50.0) (p=.013)1**
**Accommodation type**				
Owner occupied/mortgage	99 (81.8)	76 (81.7)	95 (79.8)	79 (82.3)
Rented private landlord	7 (5.8)	6 (6.5)	11 (9.2)	5 (5.2)
Rented Department of Housing	8 (6.6)	7 (7.5)	5 (4.2)	7 (7.3)
Other arrangement	7 (5.8)	4 (4.3)	8 (6.7)	4 (4.2)
**Employment status**				
Retired from paid work	48 (41.0)	33 (35.5)	49 (42.6)	45 (46.9)
Employed	33 (28.2)	25 (26.9)	42 (36.5)	31 (32.3)
Unable to work due to long-term illness/disability	12 (10.3)	12 (12.9)	7 (6.1)	7 (7.3)
Looking after home/family	11 (9.4)	7 (7.5)	8 (7.0)	6 (6.3)
Unemployed and looking for work	8 (6.8)	2 (2.2)	3 (2.6)	1 (1.0)
At school or in full-time education	1 (0.9)	0 (0)	0 (0)	0 (0)
Other	4 (3.4)	4 (4.3)	6 (5.2)	3 (3.1)
SEIFA score2**				
1 -2	10 (10.6)	7 (7.5)	9 (7.8)	6 (6.3)
3-4	5 (5.3)	4 (4.3)	3 (2.6)	3 (3.1)
5-6	7 (7.4)	6 (6.5)	13 (11.3)	10 (10.4)
7-8	7 (7.4)	5 (5.4)	14 (12.2)	11 (11.5)
9-10	65 (69.1)	49 (52.7)	76 (66.1)	59 (61.5)

1 At follow-up a statistically significant number of participants in the control group reported being born in a country other than Australia compared to intervention participants.

2 ** Socio-Economic Indexes for Areas (SEIFA) have been constructed by the Australian Bureau of Statistics from the 2006 Census of Population and Housing data. These indexes allow comparison of the social and economic conditions across Australia. Lower values indicate lower socio-economic status.

There were no significant differences between participants in the intervention or control groups related to gender, age, language spoken at home, accommodation and employment status, socioeconomic status, type, number and duration of chronic illnesses. However, more participants in the control group reported having been born outside Australia (51.2% vs 38.8%; X^2^=6.210, df=1, p=0.013) (Table 4).

### Outcomes

#### Primary hypotheses

##### Self-efficacy

Mean self-efficacy scores did not differ significantly between intervention and control groups at baseline or follow-up. There was an increase in self-efficacy score from baseline to 4 months in intervention program attenders (+0.37, t=2.315, df=60, p=0.028) but no change in the control group (Table 5). There was no difference between groups in the intention to treat analysis (Table 5), nor after adjusting for covariates (age, gender, number of chronic illnesses, country of birth and baseline score) in the multivariate analyses (Table 2).

**Table 5:** Univariate analyses for continuous outcome variables at baseline and 4 months follow-up

	**Intervention group**			**Control group**				
	**Baseline**	**4 months follow-up**		**base**	**4 months follow up**			
		**Program attenders (n=62)**	**ITT1 analysis(N=93)**		**Program attenders (N=77)**	**ITT analysis (N=96)**	**Program attenders Intervention vs Control follow-up**	**ITT Intervention vs Control follow-up**
	**Mean (SD)**	**Mean (SD) p (1-tailed)2**		**Mean (SD)**	**Mean (SD) p (2-tailed)**		**P (1-tailed)**	**P (1-tailed)**
Primary Outcomes								
Self-efficacy	6.42 (2.13)	**6.79 (2.09) p=0.028**	6.64 (2.15) p=0.094	6.72 (2.08)	6.89 (2.07)p=0.423	6.99 (2.01) p=.084	P=0.323	0.099
Diet score	5.26 (2.05)	**5.67 (2.16) p=0.048**	**5.40 (2.10) p=0.028**	4.96 (1.81)	4.79 (2.05)p=0.361	4.96 (2.06) p=0.500	**P=0.011**	0.091
Physical activity score	1.57 (.890)	**2.03(.802) p=0.000**	**1.92 (.922) p=0.000**	1.61 (.984)	1.77 (.938) p=0.122	1.77 (.921) p=0.187	**P=0.038**	0.112
Self-rated health	2.11 (.777)	2.00 (.753) p=0.073	3.07 (.912) p=0.144	2.01 (.739)	2.11 (.741)p=0.163	3.17 (.919) p=0.230	P=0.207	0.201
Health distress	1.92 (1.34)	**1.59 (1.33) p=0.002**	**1.72 (1.34) p=0.034**	2.02 (1.28)	**1.64 (1.33)p=0.001**	**1.65 (1.27) p=0.000**	P=0.414	0.295
Secondary outcomes								
Work and social adjustment	16.74 (11.70)	**14.78 (11.53)p=0.004**	**15.02 (11.74) p=0.037**	15.60 (11.68)	**13.99 (10.58)p=0.044**	**14.17(10.63) p=0.028**	P=0.345	0.127
Anxiety score	6.50 (4.34)	**5.54 (3.86) p=0.010**	6.30 (4.16) p=0.259	6.59 (3.96)	**5.55 (3.87) p=0.004**	**5.75 (3.67) p=0.000**	P=0.447	0.156
Depression score	5.59 (4.22)	5.11 (4.04) p=0.133	5.36 (4.31) p=0.144	5.84 (3.80)	**5.01 (3.57) p=0.013**	**4.70 (3.42) p=0.000**	P=0.446	0.133
GP visits	3.41 (2.73)	**2.63 (2.82) p= 0.044**	3.22 (4.33) p=0.423	3.12 (3.94)	2.42 (3.67) p=0.050	**2.69 (3.65) p= 0.057**	P=0.367	0.193

Notes:

1. Intention to treat (ITT) analysis combines follow-up data of the “program attenders” and those that withdrew prior to commencing the intervention program or during the program, that is, considering everyone that was allocated to the intervention program as being part of the trial, whether or not they attended or completed the program.

2. In view of the directionality of the research hypotheses (i.e. the results of the intervention group were expected to be better than for the control group), one-tailed tests were carried out for the intervention group.

#### Self-management knowledge and lifestyle behaviours

##### Existence of a self-management or action plan for chronic illness

57.6% of participants reported that they had a self-management plan at baseline, with no significant difference between intervention and control groups. At 4 months follow-up, a significantly greater proportion of participants in the control group (20.8%) indicated that they did not have a self-management plan compared to those in the intervention group (8.8%) (X^2^= 4.671, df=1, p=0.031).

Approximately one-third of the intervention participants with an action plan were already following their plan, with a further 10 participants intending to start their plan soon.

##### Sharing self-management plan with primary care provider post program attendance

Information concerned with willingness to share an action or self-management plan with a primary care provider post-program attendance was recorded only for the 62 intervention program attenders.

With regard to the action plan, 23 people (37.1%) were willing for the program coordinator to send it to their referring health care provider, 35 (56.5%) were not and four (6.5%) did not answer the question. Fewer were willing for it to be sent to their General Practitioner: 12 people (19.4%) were willing, 38 (61.3%) were not and 12 (19.4%) did not answer the question.

With regard to the self-management plan, 11 (17.7%) were willing for it to be sent to their referring health care provider, 41 (66.1%) were not and 10 (16.1%) did not answer the question. Only one person (1.6%) was willing for it to be sent to their General Practitioner, 51 (82.3%) were not and 10 (16.1%) did not answer the question.

14 program attenders (22.6%) indicated their intention to take their action or self-management plan to their next General Practitioner appointment; 22 (35.5%) did not intend to do so and 26 (41.9%) did not respond to the question.

##### Using a medicines list and medicine information-seeking behaviour

At baseline there was no significant difference in use of medicine lists between intervention and control participants. At 4 months follow-up more intervention participants reported keeping a medicines list (78.6% compared to 63.9% at baseline, McNemars Test, p=0.011). There was no change within the control group. After adjusting for covariates, there was no significant difference between groups at follow-up.

There was no significant difference between or within groups with regard to medicine information-seeking behaviour.

##### Change in self-management knowledge

No significant differences were found between or within groups at baseline or follow-up in mean scores for self-management knowledge (t=0.607, df=182, p=0.27) (Component factor 1).

##### Change in self-management behaviours

No significant differences were found between or within groups at baseline or follow-up related to mean stage of change in self-management behaviour scores (t=1.224, df=183, p=0.11) (Component factor 2).

#### Reported lifestyle changes

##### Smoking

Only 15 (6.0%) participants reported smoking cigarettes at baseline and 8 (4.2%) at follow-up. There was no significant change in smoking behaviour in either group.

##### Nutrition

Diet scores were not significantly different between study groups at baseline. Intervention group scores increased but those in the control group did not. At follow-up those who had attended the intervention program had significantly higher scores than those who attended the control group (t=2.315, df=131, p= 0.011) (Table 5). In ITT analysis (Table 5) and after adjusting for covariates (Table 2), there was no significant difference between intervention and control group mean scores at follow-up.

##### Alcohol

Just over 50% of intervention participants and 54.5% of control participants reported never or only consuming alcohol once a month at baseline. There was no significant change in alcohol intake in either group.

##### Physical activity

Physical activity scores were not significantly different between groups at baseline. Scores in the intervention group increased (Table 5). At follow-up those who had attended the intervention group program had significantly higher scores than those who attended the control group (t= 1.779, df=134, p= 0.038) (Table 5). In ITT analysis and multivariate analysis adjusting for covariates, there were no significant difference between intervention or control group scores at 4 months (Tables 5 and 2).

#### Perceived health status

There were no significant differences in self-rated health scores between the groups at baseline or follow-up (Table 5).

#### Secondary hypotheses

##### Work and social adjustment

Approximately 50% of each group at baseline reported “significant to severe” functional impairment as measured by their baseline scores on the Work and Social Adjustment scale.

Both groups reported a within-group decrease in mean work and social adjustment scores at follow-up (Table 5). However, no significant differences were found between the intervention and control groups at follow-up for either Program Attenders, ITT analyses (Table 5) or multivariate analysis adjusting for covariates (Table 2).

##### Levels of anxiety

There was no significant between-group difference for mean anxiety scores at baseline nor were there between-group differences in terms of change in mean anxiety scores for program completers (p=0.447) or ITT analysis (p=0.156) (Table 5). Both intervention and control groups reported a within group decrease in mean anxiety scores from baseline to follow-up for the intervention Program Attenders (t=2.650, df=55, p= 0.010), control group attenders (t=2.948, df=73, p=0.004) and control group ITT analyses (t=3.662, df=92, p <0.001) (Table 5).

##### Levels of depression

There was no significant difference in depression scores between intervention and control groups at baseline or follow-up. For both control program attenders (t=2.537, df=67, p=0.013) and control group ITT analyses (t=4.093, df=86, p < 0.001) there was a significant within-group reduction in mean depression scores at follow-up. There was no significant difference in the mean depression scores for the intervention group from baseline to follow-up (Table 5).

##### Use of health services

Participants reported a relatively low mean number of visits to an emergency department and admissions to hospital at baseline and follow-up. No significant changes were found in either group.

11.2% of participants reported a visit to a community health centre in the 4 months prior to joining the study, with no differences between groups at baseline or follow-up.

The mean number of GP visits significantly decreased in those attending the intervention program (t=1.741, df=55, p=0.044) but not in those attending the control group (Table 5). However, no significant differences were found between the intervention and control groups at follow-up for either program attenders, intention to treat analyses (Table 5) or in multivariate analysis adjusting for covariates (Table 2).

## Discussion

This study shows mixed results for the Moving On program, with some positive trends in relation to self-efficacy and self-management behaviours related to increased physical activity and healthy diet by those that attended the program.

Comparisons between the Moving On program and the Stanford and Expert Patient programs were limited by a lack of standardisation across the initiatives and their evaluations. The characteristics of participants were reported differently and different outcomes and measures were used in the evaluations. This is, in part, unavoidable given the different context, purpose and structures of the programs, but the use of standard methods in future research would make it easier to compare the impact of the group education programs within different contexts. Nevertheless, broad comparisons are made below.

Compared with the initial evaluations of the Stanford and the Expert Patient programs, Moving On participants were slightly younger but the proportion of female participants (65-70%) was similar. The Stanford program targeted arthritis, circulatory diseases and stroke in their eligibility criteria, thus a larger proportion of Stanford participants had those illnesses compared with Moving On and the Expert Patient program [5,8,18]. Illness type may influence outcomes of interest such as self-efficacy, health status and service utilisation and therefore, may account for some of the differences in findings between the studies.

The Stanford and Expert Patient program evaluations showed an increase in self-efficacy for intervention participants compared to control participants. The Moving On evaluation found that self-efficacy scores increased within the intervention group from baseline to follow-up but not between the intervention and control groups. This may in part have been a ceiling effect: Moving On participants had higher self-efficacy scores at baseline in both the intervention and control groups than participants in the Stanford and Expert Patient evaluations and so had less scope for improvement [8,18]. This may explain the small size of the improvement in mean self-efficacy scores (0.37) in the intervention participants [7]. This is further supported by the Expert Patient Program evaluation, which showed greater gain in self-efficacy in participants who had lower self-efficacy scores at baseline [21].

It is hypothesised that a positive change in self-efficacy would result in an increase in health status and a decrease in health service utilisation [22]. The Stanford program showed an increase in self-efficacy, health status and decrease in the number of visits to physicians or the emergency department. The Expert Patient Program improved self-efficacy and health status but showed no changes in service utilisation. Moving On did not lead to any change in participants’ health status but was associated with a decrease in GP visits. The proportion of participants who had more than four visits to a GP in the previous 4 months decreased by 16% in the intervention group and 6.4% in the control group. This suggests that the Moving On program may have contributed to more appropriate use of GP services. This may relate to the increase in self-efficacy or alternatively may be due to the social support and management tips exchanged by participants while attending the group sessions. This warrants further research.

The Moving On intervention group reported improved diet and physical activity scores compared to control program attenders. Improvement in diet was not found by the Expert Patient Program evaluation and not measured in the Stanford evaluation. All of the studies found an improvement in physical activity scores. The improvement in physical activity scores in the Moving On evaluation was a little surprising, given the focus on exercise in the control program. This may be because the Moving On program has modules that focus specifically on diet and physical activity: in the follow-up interviews (not reported here) many intervention participants reported having set diet and exercise goals after attending the program. This may be because these are tangible goals, which are consistent with ongoing GP recommendations or management.

All three self-management programs were associated with improvements in health distress scores. However, in the Moving On evaluation, health distress and work and social adjustment scores improved in both the intervention and control groups. This suggests either that the influences occurred independently of program participation or that both programs had an effect. This is worth noting, given that approximately 50% of each group reported significant to severe functional impairment at baseline. These perceived improvements did not appear to be related to changes in perceptions of health status, as measured by the self-rated health scale. This may suggest that they had adjusted to living with their chronic illnesses.

Other outcomes associated with the Moving On program and not evaluated by the Stanford and Expert Patient programs deserve comment. Overall, there was little difference between the two groups in the Moving On evaluation in the adoption of a range of self-management behaviours. This may reflect the stage of their chronic conditions: the average duration was nine years, during which time they may have largely adapted to living with their condition. The diversity of their conditions may also have meant that initiating some of the self-management behaviours (e.g. sleep patterns) was not relevant to all of them. This fits with the finding that at baseline some of the intervention participants were already in the action or maintenance phase of behaviour change. It is also possible that the self-management behaviours in the evaluation did not reflect the way patients managed their illnesses [23]. This is an area where further aligning program content and evaluation measures with patient needs is likely to lead to better programs and more generalisable evaluations.

More intervention participants had a self-management plan at follow-up. However, the follow-up interviews with participants showed that patients had different ideas about what a self -management plan was and what its purpose was. This needs further study and more sensitive measurement.

Self-management plans were sent to primary care providers with participants’ consent. This was an attempt to encourage GPs to take an active role in supporting patient self-management after completion of the program, thereby addressing the disconnection between self-management education programs and primary health care providers [24]. This is important as GPs are encouraged to take an active role in self-management support [2]. The relatively small number of participants who agreed to share their self-management plan with their GP was surprising. It is likely that patient-GP collaboration regarding self-management plans is complex and influenced by the patient-provider relationship, their views of the role of primary care providers and their understanding of self-management among other factors. How patients conceptualise self-management and their views of self-management plans is the subject of an ongoing secondary study associated with this RCT.

Anxiety scores improved for participants in both the Moving On intervention and control groups, but only the control participants improved their depression scores. The reasons for this are unclear. It may have been due to the positive effects on well-being associated with exercise programs [25]; however, the reported frequency of physical activity was greater in the intervention group.

The study had a number of limitations. Although post hoc sample calculations suggest that there were adequate numbers of participants to detect change in primary outcomes, a high proportion of participants did not complete either the intervention or control program. This not only reduced the power but confounded the between-group analyses. Thus, although there were significant changes in self-efficacy scores for those completing the intervention program and not in those completing the control program, there was no difference between groups as allocated, especially when adjusted for covariates.

A number of factors contributed to the high attrition rate (40.5%), which was higher than that reported for the Stanford program (17.8%) [5,18] and the Expert Patient Program (17.1%) [8]. This may, in part, arise from the design of the study. Using an active control program instead of the wait-list control methods used by the other programs meant that participants did not know which arm of the study they would be allocated to. Some people who withdrew after allocation to the intervention program said that they had hoped to be allocated to the light physical activity program. Allocating patients to interventions after enrolment made it difficult and more complex for health care providers to explain the study to patients and provide sufficient information about both programs. The uncertainty of allocation may have made some patients reluctant to commit to the study, resulting in a high non-enrolment rate. However, the strength of this design was that it makes it easier to attribute any changes to the impact of the specific intervention program, rather than simply being an effect of participating in a group program at all.

Although GPs and other health care providers were generally positive about the referral process, they indicated in follow-up interviews that excluding patients aged over 75 or not proficient in English limited the number of potential participants and so the ultimate size of the study. However, these conditions are likely to have strengthened the program by excluding some who would not be able to benefit because of subtle cognitive impairment or with difficulty in understanding English.

Both the Stanford and Expert Patient programs focused on primary outcomes over a six month period and the Stanford program was subsequently evaluated over 12 and 24 months. In comparison, the Moving On evaluation focused on changes in self-efficacy, health status and self-management behaviours over a four month period. A longer follow-up period (6–12 months) would be desirable.

Overall, participants in the Moving On evaluation were found to be low users of health services other than general practice and the study had insufficient power to detect small changes. Therefore, the findings associated with health service use need to be interpreted with caution.

Finally, the sample population of Moving On was found to be relatively homogeneous, the majority being Australian-born, English-speaking people who owned their own home in a high socio-economic index locality and who were either currently employed or retired from paid employment. Coverage of disadvantaged populations was limited. These findings are similar to other international evaluations of chronic disease self-management programs [24]. This has implications for the applicability of the study findings and future implementation of Moving On to population groups not represented in the sample.

## Conclusions

Overall, the Moving On program attenders demonstrated positive trends in self-efficacy and some self-management behaviours. However, non-completion rate in both arms of the study, combined with low enrolments, reduced the study power and diluted the impact of the intervention. Further research is needed to evaluate the impact and cost effectiveness of the program, over a longer period than was possible in this study and with more diverse populations.

## Abbreviations

SHCI: Sharing Health Care Initiative; COAG: Council of Australian Governments’; RCT: Randomised Controlled Trial; HADS: Hospital Anxiety and Depression Scale; ITT: Intention to treat analysis; AHS: Area Health Service

## Competing interests

Diana Aspinall and Nicholas Manolios are Associate Investigators and Directors on the Board of Arthritis NSW. Karen Filocamo is an Associate Investigator and the CEO of Arthritis NSW. Eloise Milthorpe is employed by Arthritis NSW as a Senior Project Officer. The authors declare that they have no competing interests.

## Authors’ contributions

AW contributed to the conception and design of the study, data collection, analysis and interpretation and developed the draft manuscript. MH contributed to the conception and design of the study, data analysis and interpretation and development of the manuscript. LB contributed to the conception and design of the study and development of the manuscript. DA and KF contributed to the design of the study. UWJ performed the block randomizations and provided statistical advice during analysis and provided conceptual input into the draft manuscript. EM, NM, TW provided conceptual input into the draft manuscript. EM was also engaged in data collection and proof reading the manuscript. All authors read and approved the final manuscript.

## Pre-publication history

The pre-publication history for this paper can be accessed here:

http://www.biomedcentral.com/1472-6963/13/90/prepub
